# Aspects of vincristine-induced neuropathy in hematologic malignancies: a systematic review

**DOI:** 10.1007/s00280-019-03884-5

**Published:** 2019-06-18

**Authors:** Marie Lindhard Madsen, Hanne Due, Niels Ejskjær, Paw Jensen, Jakob Madsen, Karen Dybkær

**Affiliations:** 1 Department of Hematology, Aalborg University Hospital, Sdr. Skovvej 15, 9000 Aalborg, Denmark; 2 Department of Clinical Medicine, Aalborg University, Sdr. Skovvej 15, 9000 Aalborg, Denmark; 3 Steno Diabetes Center North Denmark, Aalborg University Hospital, 9000 Aalborg, Denmark; 4 Clinical Cancer Research Center, Aalborg University Hospital, Sdr. Skovvej 15, 9000 Aalborg, Denmark

**Keywords:** Vincristine, Vincristine-induced neuropathy, Neurotoxicity, Hematologic malignancies, Biomarkers

## Abstract

**Purpose:**

Vincristine is widely used as anticancer therapy for a variety of hematological malignancies. The treatment is limited by progressive vincristine-induced neuropathy, possibly including both peripheral sensory and motor nerves, autonomic nervous functions, and the central nervous system. This dose-limiting side-effect can diminish quality of life and, furthermore, cause discontinuation of vincristine treatment. The present review elucidates the current knowledge regarding vincristine-induced neuropathy in hematologic malignancies, focusing on neuropathy assessment, clinical and molecular predictive markers, drug–drug interference, prevention, and treatment.

**Methods:**

This review is conducted by a systematic search strategy for the identification of relevant literature in the PubMed and Embase databases.

**Results:**

No clinical parameters displayed convincing potential as predictors of vincristine-induced neuropathy; however, preexisting neuropathy was consistently reported to be associated with an increased risk of neurotoxicity. In contrast, molecular markers, including polymorphisms in genes involved in the pharmacodynamics and pharmacokinetics of vincristine, displayed great potential as predictive markers of neuropathy incidence and severity. Furthermore, antifungal drugs, such as itraconazole and voriconazole, decrease the metabolism of vincristine and consequently lead to severe neuropathy when co-administered with vincristine, underscoring why fluconazole should be the antifungal drug of choice.

**Conclusion:**

Reports from the 71 included studies clearly emphasize the lack of consistency in neuropathy assessment, grading systems, and reporting, making it difficult to interpret results between studies. Thus, truer clinical and molecular markers could emerge if the consistency of neuropathy detection and reporting increases by the use of conventional standardized neuropathy assessment tools and grading scales.

**Electronic supplementary material:**

The online version of this article (10.1007/s00280-019-03884-5) contains supplementary material, which is available to authorized users.

## Introduction

Vincristine is a chemotherapy drug belonging to the group of vinka alkaloids, which also includes vinblastine and vindesine, which has been widely used since its approval in 1963 [1]. The antimitotic drug is used in the treatment of several solid tumors and hematologic malignancies, including breast cancer, non-Hodgkin’s lymphomas (NHL), and leukemia [2]. Vincristine exerts its anti-neoplastic effect by inhibiting polymerization of tubulin and incorporation into microtubules, which prevents mitotic spindle assembly, leading to extension of mitosis and thereby apoptosis [3, 4].

The dose-limiting side effect of vincristine is neurotoxicity, which may lead to severe peripheral sensory and motor neuropathies affecting quality of life (QOL), treatment delay, and vincristine substitution or discontinuation. The impact of adapted use of vincristine on outcome is debatable [5–7]. Vincristine interferes with microtubules, which are critical components of nerve axons functioning as tracks for vesicle-mediated transport, and leads to axonopathy that manifests slowly and progressively [8]. Vincristine-induced neuropathy (VIN) spans a broad spectrum of dysfunctions that fall into three categories: sensory, motor, and autonomic neuropathy (Table 1). The most common is peripheral sensory and motor nerve neuropathy characterized by numbness, paresthesia, impaired balance, weakened tendon reflexes, and altered gait [9]. Autonomic dysfunctions includes constipation, paralytic ileus, urinary retention and orthostatic hypotension [10, 11]. Furthermore, several cranial nerve palsies and some central nerve system (CNS) toxicities have been reported (Table 1) [12–27], even though vincristine poorly penetrates the blood–brain barrier [28]. Although neurotoxicity is widely recognized as a common side-effect of vincristine treatment in hematologic neoplasms, little is known about the true incidence, short- and long-term manifestations and severity due to lack of consistency in detection, definition, and reporting.

**Table 1:** Types of vincristine-induced neuropathy

Type	Definition	Symptoms	References
Sensory neuropathy	Sensory nerve damage	Paresthesia in form of numbness, tingling and pricking. Pain, impaired vibration/touch sensitivity/temperature recognition	[9, 92]
Motor neuropathy	Motor nerve damage	Motor weakness, walking difficulties, muscle cramps, weakened tendon reflexes and fine motor skills	[92]
Autonomic neuropathy	Autonomic nerve damage	Constipation, ileus, urinary retention, incontinence, hypotension	[11, 92]
Optic neuropathy	Cranial nerve II damage	Blurred vision, color vision deficiency, transient/permanent blindness	[12, 13]
Oculomotor nerve palsy	Cranial nerve III damage	Ptosis, ophthalmoplegia	[14–16]
Abducens nerve palsy	Cranial nerve VI damage	Ptosis, strabismus, ocular muscle paresis, diplopia	[17, 18]
Facial nerve palsy	Cranial nerve VII damage	Limited movement of facial muscles and jaw	[19]
Acoustic nerve palsy	Cranial nerve VIII damage	Hearing loss	[20]
Ototoxicity	Cochlear damage	Decrease in frequencies, decrease of contralateral suppression amplitudes	[21]
Hypoglossal nerve palsy	Cranial nerve XII damage	Loss of tongue movement	[19]
Vocal cord palsy	Laryngeal nerve damage	Stridor, respiratory distress, persistent cough	[22, 23]
Encephalopathy/PRES	Cerebral dysfunction	Disorientation, hemiplegia, global aphasia, seizures	[24, 25]
SIADH	Cerebral axonal swelling	Hyponatremia, seizures, mental changes	[26, 27]

*PRES* posterior reversible encephalopathy syndrome, *SIADH* syndrome of inappropriate antidiuretic hormone secretion

Overall survival for adult hematologic cancer patients has improved during the past decades due to new treatment options, and more than 80% of children with acute lymphoblastic leukemia (ALL) are now long-term survivors [29]. This therapeutic success, however, comes with the cost of more people experiencing early- and late-onset adverse effects, consequently affecting the recovering patient’s QOL, which is especially important in children with a long expected lifespan after treatment. Although the intensity of the symptoms may not be extensive, the inconvenience is not correlated, and QOL can be greatly impaired [30]. Given the increasing numbers of cancer survivors, the clinical significance of chemotherapy-induced neuropathy is increasing; consequently, clinical and molecular risk predictors, prevention and treatment options, and measuring methods are urgently warranted. In this paper, we systematically review parameters related to vincristine-induced neurotoxicity in hematologic patients, and we discuss their importance.

## Methods

This review was completed according to the Preferred Reporting for Systematic Reviews and Meta-analyses (PRISMA) Guidelines [31].

### Search strategy and study selection

PubMed and Embase databases were systematically searched for current literature on VIN in patients with hematologic malignancies. Supplementary Table 1 outlines the search strategy. The search was performed on 16 July 2018 and identified 1949 articles after removal of duplicates, which subsequently were manually screened based on title and abstract by two independent individuals. Articles not focusing on hematologic malignancies or vincristine-induced neuropathy were excluded as were reviews, conference abstracts, nonhuman studies, and data not published in English. The remaining articles were assessed for eligibility by full-text screening, and 71 articles were included in this systematic review after applying the same exclusion parameters (Supplementary Fig. 1).

## Results

A total of 71 articles were included in this systematic review (Supplementary Fig. 1). Of these articles, 13 investigated VIN measuring methods [9, 32–43], while 11 records investigated clinical risk predictors for VIN development [44–54]. Fifteen records studied molecular risk parameters [5, 55–68]. In addition 12 records described interactions between vincristine and other drugs, mainly antifungal triazoles [1, 69–79], while 8 records addressed prevention and treatment options [10, 19, 80–85]. The remaining 12 records were of mixed character and included long-term effects, assessment of QOL, and dose-dependent VIN studies [30, 86–96].

The 71 articles include studies of VIN in both pediatric and adult hematologic malignancies, including ALL, diffuse large B cell lymphoma, follicular lymphoma, multiple myeloma, Burkitt lymphoma, anaplastic large-cell lymphoma, and Hodgkin’s lymphoma. The reported frequency of vincristine-induced neurotoxicity in these studies varies tremendously from approximately 100% [34] to 10% [48] depending on patient exclusion criteria, vincristine doses and number of treatment cycles, and the methods of obtaining neuropathy information.

### Methods for VIN measuring

Numerous methods for assessment of neuropathy are used in different studies. There is no gold standard, making it difficult to compare studies. This lack of a standard is most likely the reason why the reported incidence of neuropathy in patients treated with vincristine varies so significantly. In 1981, the World Health Organization (WHO) initiated a set of recommendations on standardized approaches to recording baseline data, reporting of treatment, reporting of response, and grading of acute and subacute toxicity to assess the problem that it is difficult for investigators to compare their results with those of others [97]. WHO’s peripheral neuropathy score comprises 5 grades (0–4); noteworthy, only two of the included studies applied WHO’s neurotoxicity score [5, 65].

In 2003, the National Cancer Institute of the National Institutes of Health released the Common Terminology Criteria for Adverse Events (CTCAE), which was revised from a widely used neuropathy grading scale, named the Common Toxicity Criteria, that was developed in 1984 [98]. CTCAE is still occasionally used today [67]. However, as it was shown to underestimate both incidence and severity of neuropathy, several scores have been made to overcome this problem, including the Total Neuropathy Score (TNS) [34, 99]. TNS consists of ten items that are both subjective and objective; however, the TNS was found to be too burdensome and time-consuming for application in everyday clinical practice and consequently reduced into a clinical version (TNSc), a reduced version (TNSr), and a neuropathy score that quite often used in pediatric patients, called the Pediatric modified Total Neuropathy Score (Ped-mTNS) [32]. The Ped-mTNS consists of the first seven items of TNS and was shown to be a reliable and valid measure of VIN in school-aged children [33]. Compared with Ped-mTNS, CTCAE version 3.0 failed to identify sensory neuropathy in 40% of subjects and significant motor neuropathy in 15% when applied on children treated for ALL, lymphoma or non-CNS solid tumors [34]. Thus, the detection method itself greatly influences the result, illustrating the need to introduce and unify detection and quantification of VIN.

A study examined the validity, reliability and clinical feasibility of several VIN measures in ALL children [35]. The study mostly concentrated on a variation of TNS, referred to as Total Neuropathy Score-Pediatric Vincristine (TNS-PV), which was found useful for measuring VIN in children over the age of 5 years; additionally, some of the items were responsive to change over time. Furthermore, they tested CTCAE version 4.0 and the Balis grading scale, which both presented a risk of underestimating VIN. The FACES pain scale was feasible for pain severity quantification in children of all ages. In addition to the previously established measurements, they developed a simple test V-Rex consisting of the two most responsive items, vibration and reflex, which is better than standard grading scale methods according the study [35].

Symptoms of VIN are mostly subjective; thus, self-reported data could be preferable in VIN assessment. The European Organization for Research and Treatment of Cancer (EORTC) developed a questionnaire intended to supplement the core quality of life questionnaire to assess chemotherapy-induced neuropathy (QLQ-CIPN20) [42]. The 20-item questionnaire includes information on symptoms and functional limitations, and comparison to CTCAE in two large datasets revealed a strong correlation between patients with high CTCAE grade and QLQ-CIPN20 score, documenting the validity of self-reported data in terms of chemotherapy-induced neuropathy [43].

Interestingly, studies have shown a difference between children and adults in the type of nerves that are the most affected with a predominance of sensory neuropathy in adults [90, 100, 101]. Electrodiagnostic examinations, including nerve conduction of different nerves, T-reflex measurement and needle electromyography, showed a electrophysiological and clinical motor predominance in children aged 1–17 years [39], which is consistent with other studies using nerve conduction and somatosensory evoked potentials [9, 38, 41, 86]. Furthermore, a study demonstrated that measurements of compound muscle action potential amplitude on the median and/or peroneal nerve could objectively grade VIN in children [9]. In addition to sensory and motor, the third neuropathy modality is autonomic. Heart rate variability, reflecting a vagal nerve lesion, is useful to detect and quantify autonomic cardioneuropathy in children diagnosed with ALL [36]. Furthermore, a study used The Michigan Autonomic Symptoms Survey, but interestingly no abnormalities in autonomic functions were found [40].

Many new neurotoxicity scores have continuously been developed. Of these scores, some focus on objective findings, while others are of more subjective character. Although CTCAE still develops new versions, it seems to have been outdated by newer approaches. Despite findings suggesting motor predominance in children and sensory predominance in adults, no consensus exists on the best methods of obtaining VIN information in hematological patients of different age.

### Clinical predictors

Although occurrence of neurotoxicity is high, little is known about risk factors for developing neuropathy during vincristine treatment. A consensus about possible risk factors is lacking, and numerous parameters are only studied and reported in a limited number of studies with relatively small study populations. The dose-limitation of vincristine is 1.4 mg/m^2^ in each cycle with a maximum of 2 mg per cycle, and higher doses exert greater toxicity and questionably result in better treatment outcome [91, 92, 102]. The most consistent observation is that the severity of neuropathy increases with accumulated vincristine dose [49, 92, 103]. Furthermore, several studies report advanced age in pediatric patients as a clinical risk factor [61, 69, 73]. However, some studies do not find this correlation in pediatric or adult patients [44, 49] and others even suggest the opposite for both groups [54, 88, 95].

It has been suggested that neuropathy incidence could be disease dependent given that a significantly higher incidence in patients suffering from lymphoma is observed compared with nonlymphoid cancer patients (e.g., leukemia, breast cancer, malignant melanoma) [88]. Fourteen out of 23 patients with lymphoma and 5/37 patients without lymphoma developed neuropathy despite comparable dosage [88], and concordant observations were made by others [46]. A higher percentage of lymphoma patients have elevated levels of serum alkaline phosphatase, which significantly prolongs elimination of vincristine and results in longer vincristine exposure and associated toxicities [46]. Furthermore, the results from this study indicate that area under the vincristine plasma concentration time curve and elevated serum alkaline phosphatase are predictors of VIN [46]; however, the latter observation is contradicted in a study of 26 patients reporting that serum alkaline phosphatase is not important [92].

Several factors have been suggested as possible predictors for VIN. A study from 2010 aimed to identify predictors for chemotherapy-induced peripheral neuropathy, including vincristine, in 52 patients [49]. It was discovered that the number of chemotherapy cycles was a predictor of VIN, whereas age and coadministration with nonsteroidal anti-inflammatory drugs did not correlate with neuropathy incidence and severity. Consistently, Anghelescu et al. observed no difference in age between ALL patients with or without neuropathy development [44]. No differences in sex, BMI group, initial leukocyte count, ALL immunophenotype, DNA index, or different genetic translocations were noted. The only significant clinical predictive variable observed was white non-Hispanic race [44].

Another study focused on the potential effect of micronutrient deficiency on VIN by measurement of serum vitamin E, vitamin B_12_ and folate together with nerve conduction studies at a mean 20 months after the last vincristine injection; however, no significant association was observed [45]. Furthermore, the impact of liver dysfunction was examined in two independent studies, where one recommended reduced vincristine dosage [46] whereas the other did not [88].

Despite the fact that some records mention diabetes mellitus as a risk factor for developing VIN [103–105], thorough studies are lacking. Patients with diabetes are at risk of diabetic neuropathy; therefore, it could be logical to assume that diabetic patients are at higher risk for VIN. Noteworthy, diabetic neuropathy is a long-term risk of diabetes; therefore, it is important to be aware of the difference between diabetes as a risk factor and diabetic neuropathy as a secondary risk factor.

Hyperglycemia is a common side-effect of corticosteroid therapy that is often noted with vincristine treatment and potentially leads to diabetes [106]. The effect of hyperglycemia was studied in 278 patients with ALL during induction chemotherapy regimens including vincristine [48]. Hyperglycemia was defined as glucose level ≥ 200 mg/dL on ≥ 2 determinations, and the study included 20 patients (7%) previously diagnosed with diabetes. Statistically significant differences in peripheral neuropathy were not noted between 103 patients with hyperglycemia and the 175 patients without; furthermore, a subanalysis of patients with previously diagnosed diabetes or elevated baseline blood glucose did not reveal any differences. Consistently, no difference in neuropathy incidence was found when comparing diabetic patients requiring diabetic medications with nondiabetic patients [54].

Furthermore, the consequences of hyperglycemia in elderly patients were examined and included 162 patients with NHL treated with vincristine among other chemotherapeutic drugs [47]. The authors found that baseline hyperglycemia, stage, creatinine clearance and hyperglycemia during chemotherapy was associated with grade 3–4 toxicity of 18 symptoms, including neuropathy, diarrhea, fatigue and pneumonia, without separate and focused analysis of the individual symptoms.

The risk of severe neurotoxicity due to other forms of pre-existing diseases than diabetes is more substantiated. Care should be taken when administrating vincristine to Charcot-Marie-Tooth disease (CMT) patients, the most common hereditary neuropathic disease. A clinical challenge is undiagnosed cases of CMT, and seeking information about family history of CMT before initiating chemotherapy with vincristine could possibly prevent patients from severe neuropathy given that pediatric ALL often is diagnosed before the age of 10 and CMT after the age of 10 [51]. Pediatric and adult cancer patients are at risk of undiagnosed CMT [50]. Even small doses of vincristine that are normally not associated with neuropathy can cause exaggerated and irreversible neuropathy in these patients [50]. Another form of neuropathy presenting clinical problems is the autoimmune Guillain–Barré syndrome (GBS). Acute onset GBS can be difficult to differentiate from VIN, and this differential diagnoses is important to have in mind as GBS can be treated with immunoglobulins. Uncertainty still remains whether GBS and VIN can exaggerate one another [52, 53].

This section clearly emphasizes the inconsistency between studies; however, clinical parameters, including white non-Hispanic race and preexisting neuropathy, such as CMT, are consistently reported in more than one study to predict increased risk of VIN. When assessing the currently available literature, preexisting diabetes does not increase the risk of VIN. Hyperglycemia presenting during induction chemotherapy or later in the course of chemotherapy also does not increase the risk of VIN.

### Molecular predictors

The hepatic cytochrome P450 3A (CYP3A) enzyme subfamily is the most important system for drug metabolism. Vincristine is primarily metabolized by CYP3A4 and CYP3A5, of which the latter is considerably more effective [107]. Given that 60% of African–Americans express the CYP3A5 enzyme compare with 33% of Caucasians [108], several studies have investigated race-specific genotypes and tested whether African–Americans metabolize vincristine more effectively, resulting in lower vincristine exposure and associated toxicities (Table 2). A retrospective study analyzed 92 Caucasian and 21 African–American pediatric ALL patients and identified VIN symptoms in 35% of Caucasians compared with 5% of African–Americans [57]. Additionally, Caucasians experienced more severe vincristine-associated neuropathy and had more reductions in total doses. However, it is important to highlight that race is used as a surrogate for the *CYP3A5* genotype, and no genotyping was conducted.

**Table 2:** Molecular predictors of vincristine-induced neuropathy

Polymorphism	Outcome	Race	No. of patients	Genotyping	Neuropathy assessment	References
Race used as surrogate for CYP3A5	VIN in 35% of Caucasians and 5% of African–Americans	92 Caucasians 21 African–Americans	113 children (ALL)	Not performed	Not reported	[57]
	↑ VIN severity in Caucasians					
	Dose reduction in 4% of Caucasians and 0.1% of African–Americans					
CYP3A5*3	82% of non-African–Americans 25% of African–Americans	31 African–Americans 502 Other	533 children (ALL)	Pyro-sequencing	CCG toxicity criteria	[55]
CYP3A4*1B CYP3A5*3	NS					
CYP3A5*6	NS					
	VIN in 81% of Caucasians and 77% Africans–Americans	37 Caucasians 17 African–Americans	54 children (ALL)	RT-qPCR	Balis Pediatric Scale	[64]
CYP3A5*3	67% of the patients: 27 Caucasians, VIN in 82% 8 African–Americans, VIN in 75%					
CYP3A*1/*1 CYP3A*1	91% of patients CYP3A5 high expressers	Kenyan	78 children (Leukemia/ Lymphoma: 44 solid tumors: 34)	OpenArray RT-qPCR	NCI-CTCAE, Balis Pediatric Scale, Faces Pain Scale, Pediatric Neuropathic Pain Scale, TNS	[67]
CYP3A5*3	CYP3A5 low expressers Higher vincristine plasma dose					
	No difference in VIN					
CYP3A1*/*1	1% of patients	Turkish	115 children (ALL: 72 solid tumors: 43)	RFLP	NCI-CTCAE	[66]
CYP3A5*1/*3	15% of patients VIN in 18%					
CYP3A5*3/3*	84% of patients VIN in 22%					
CYP3A5*6 CYP3A*7	0% of patients					
CYP3A5*3/*3	82% of patients (CYP3A5 low expressers) ↑ VIN incidence + severity ↑ dose reductions	105 Caucasians 1 African–American 1 Asian	107 children (preB ALL)	RT-qPCR	NCI-CTCAE	[59]
CYP3A5*1/*3	18% of patient (CYP3A5 expressers) ↓ VIN incidence + severity					
CYP3A5*3	↑ Risk of VIN	44 black, 167 white, 1 Asian, 28 other	240 children (ALL)	Not specified	NCI-CTCAE	[56]
miR-3117*	↓ Risk of VIN Potentially affects regulation of drug transporters ABCC1 and RALBP1	Spanish	175 children (preB ALL)	GoldenGate Genotype Assay	WHO Peripheral Nervous System toxicity Criteria	[5]
miR4481*	↑ Risk of VIN Involved in peripheral nerve regeneration					
	VIN in 27% of patients	209 European 43 African 2 Asian 44 Hispanic 23 Other	321 children (ALL)	GeneChip 500 K array or SNP 6.0 array	NCI-CTCAE	[61]
CEP72*	16% of patients VIN in 56%, ↑ risk and severity					
CEP72*	31% of cases and 10% of controls. CEP72* cases: ↑ VIN	Cases: 3 Hispanic, 38 Non-Hispanic, 7 unknown. Control: 4 Hispanic, 41 Non-Hispanic, 3 Unknown	48 adults with VIN (Cases) 48 adults without VIN (Controls) (ALL)	RT-qPCR	NCI-CTCAE	[62]
	25.4% VIN in early phase 4.2% VIN in late phase	Spanish	142 children (B-ALL)	PCR	NCI-CTCAE	[63]
CEP72*	No association to neurotoxicity during the early phase of treatment					
CEP72*	↑ Incidence of VIN Detected in 16% of VIN cases and in 5% without VIN	78% European	224 children (ALL)	RT-qPCR	NCI-CTCAE	[68]
ABCC1* SLC5A7*	↑ VIN severity ↑ VIN severity			Custom Infinium Panel		
ACTG1*	↑ VIN severity grade	Caucasian	339 children (ALL)	Sequenom Genotyping	NCI-CTCAE	[60]
CAPG*	↑ VIN severity grade					
ABCB1*	↓ Risk of VIN					
ABCC2*	↑ VIN severity grade	Spanish	152 children (B-ALL)	GoldenGate Genotyping Assay	WHO Peripheral Nervous System toxicity Criteria	[65]
GLI1*	Early-onset VIN in 4% ↑ VIN	Not specified	250 adults (MM)	Custom-build targeted genotyping (3404 SNPs)	NCI-CTCAE	[58]
ABCC1*	Late-onset VIN in 7% ↑ VIN					

*ALL* acute lymphoblastic leukemia, *NCI-CTCAE* National Center Institute Common Terminology Criteria for Adverse Events, *NS* no significance, *MM* multiple myeloma, *RFLP* PCR-Restriction Fragment Length Polymorphism, *TNS* total neuropathy score

The most common genetic germline polymorphisms are CYP3A4*1B, CYP3A5*3, and CYP3A5*6, of which the latter two variants induce splice variants and protein truncation leading to substantially decreased expression of CYP3A5 in the liver [108]. Increased incidence of VIN has been observed in patients who expressed CYP3A5*3; although the percentage of African–Americans expressing CYP3A5*3 was greater than Caucasians, the difference did not reach statistical significance potentially due to the small sample size [64]. Furthermore, a higher incidence of neuropathy was detected in Caucasians (81%) than African–Americans (77%); however, substantially higher frequencies were noted for both ethnicities compared with the study by Renbarger et al. [57] (Table 2).

CYP3A variants were determined by others without focusing on ethnicity [55, 56, 59, 66, 67] (Table 2). The CYP3A5 genotype was studied in 78 Kenyan children with different cancer diagnoses [67]. Seventy-one of the 78 subjects (91%) were homo- or heterozygous for the CYP3A5*1 allele, which are phenotypic identical and lead to high expression of CYP3A5. Children with genotypes causing low CYP3A5 expression had significantly higher detectable vincristine levels in plasma compared with high expressers. Regardless of several neuropathy assessment tools, minimal neuropathy was detected, and no difference in neuropathy incidence or severity was observed despite differences in vincristine plasma concentrations. Noteworthy, Kenyan children experienced negligible VIN compared with US children despite receiving at least 33% more vincristine at baseline due to protocol-specific dosing [67], indirectly supporting the observations of race and CYP3A genotype.

Genotyping of 105 Caucasian children with ALL revealed that 82% of the patient are low CYP3A5 expressers (CYP3A5*3 genotype) with a higher incidence and severity of vincristine-induced side effects and more reductions of vincristine compared with CYP3A5 expressers [59]. Thus, the CYP3A5*3 genotype causes increased vincristine exposure, leading to higher neuropathy incidence and severity grade; however, information on clinical impact was not reported.

Studies to date have focused on genetic variants of genes involved in the pharmacokinetics of vincristine. Other biological processes can also affect VIN; hence, polymorphisms in microRNAs regulating vincristine-related genes have been examined [5]. A mutation in the seed region of miR-3117 was detected that could affect binding to the drug transporter genes ABCC1 and RALB1, which are predicted to be targets of miR-3117 in at least six prediction databases and consequently affect the regulation and expression of the targets. In addition, they identified a mutation causing alteration of the secondary structure of miR-4481a mutation, which is potentially involved in peripheral nerve generation. Consistently, polymorphisms in ABCC1 and ABCB1 drug transporters correlate with neurotoxicity, which also applies to ACTG1 and CAPG1 polymorphisms that are targeted upon vincristine treatment [60, 68]. By dividing VIN into early- and late-onset, it was observed that early-onset neurotoxicity was associated with upregulation of genes and SNPs in genes involved in cell cycle and proliferation, whereas late-onset neurotoxicity was characterized by polymorphisms in genes involved in absorption, distribution, metabolism and excretion [58].

A study was conducted for the identification of genetic germline variants associated with the occurrence and severity of VIN in 321 pediatric ALL patients using Affymetrix GeneChip 500 K or SNP 6.0 array [61]. They identified a SNP in the promoter region of CEP72 that encodes a centrosomal protein essential for microtubule formation, which is directly involved in the mechanism of action of vincristine. This mutation introduces a binding site for transcriptional repression leading to lower expression of CEP72, which causes microtubule instability and increased vincristine sensitivity [61]. The CEP72 variant was observed in 50 of 321 pediatric ALL patients, which had a significantly higher incidence and severity grade of neurotoxicity that was consistent with findings of others [68]. Furthermore, they examined the variant in adult ALL patients using a case–control setup with 48 patients who developed VIN and 48 who did not, which confirmed the high incidence of neurotoxicity in CEP72 variant patients [62]. Consistent with these findings, the potential of the CEP72 variant as a marker of VIN was investigated in Spanish ALL-diagnosed children; however, no association was found [63]. The inconsistency between studies could be due to population differences and the fact that the studies by Diouf et al. [61] focused on neuropathy in the later phase of treatment compared with the early phase in the study by Gutierrez-Camino et al. [63]. As no association was found, they conducted another study analyzing SNPs in 8 genes and 13 miRNAs involved in vincristine pharmacokinetics, which revealed strong association between neurotoxicity and polymorphisms in ABCC2 [65].

Several genes involved in the pharmacokinetics and pharmacodynamics of vincristine display potential as predictive markers of VIN risk. Thus, genotyping can be useful to guide individualized treatment to maximize the therapeutic benefit and avoid unnecessary toxicity even if the role of race and early/late VIN remains incompletely characterized.

### Drug–drug interference

Concomitant administration of drugs always carries the risk of drug–drug interaction. This effect can delay, decrease or enhance the absorption or metabolism of either drug, consequently affect the drug action and cause adverse effects. A major cause of morbidity and mortality in patients with hematological malignancies treated with immunosuppressive protocols are invasive fungal infections [70]. Antifungal triazole drugs, such as fluconazole, itraconazole and voriconazole, are the main agents for prophylaxis and treatment [1], and itraconazole is preferred given its broader spectrum of activity, especially against *Aspergillus* infections [75]. Unfortunately, the combination of vincristine and itraconazole has proven unfortunate (Table 3).

**Table 3:** Drug–drug interactions with antifungal drugs

Drug	Dose	Diagnosis	No. of patients	Neurotoxic outcome	References
Fluconazole	4 mg/kg/day max 200 mg/day 5 patients: 300–400 mg/day	ALL	197 children	NS	[69]
Fluconazole	4 mg/kg/day	ALL	31 children	NS	[73]
Fluconazole Voriconazole Fluconazole and voriconazole	Not stated	ALL (*n* = 114) HL (*n* = 16)	130 patients < 22 y/o	NS	[71]
Fluconazole	8 mg/kg/day iv (*n* = 42)	ALL	136 cases	NS	[1]
Itraconazole	5 mg/kg/day os (*n* = 44)			VIN, CNS, autonomic	
Voriconazole	8 mg/kg/day capsules (*n* = 6)			VIN severity: Itraconazole > voriconazole	
Fluconazole	5 mg/kg/day (*n* = 1)	ALL	20 children	NS	[76]
Itraconazole	5 mg/kg/day (*n *= 16)			VIN, CNS, SIADH, ileus	
Voriconazole	14 mg/kg/day iv (*n* = 3)			VIN	
Itraconazole	400 mg/day capsules	ALL	14 patients 16–29 y/o	Severe VIN, ileus	[72]
Itraconazole	5 mg/kg/day capsules	ALL (*n* = 7) B-cell NHL (*n* = 1) T cell NHL (*n* = 1)	9 children	VIN, autonomic, seizures, SIADH	[75]
Itraconazole	200 mg/day capsules	ALL	2 patients 19 and 55 y/o	Autonomic, SIADH	[74]
Itraconazole	200 mg/day os	ALCL (*n* = 1) DLBCL (*n* = 3) FL (*n* = 1) unspecified lymphoma (*n* = 2)	7 adults	Severe VIN, ileus	[70]

*ALCL* anaplastic large cell lymphoma, *ALL* acute lymphoblastic leukemia, *DLBCL* diffuse large B-cell lymphoma, *FL* follicular lymphoma, *HL* Hodgkin’s lymphoma, *iv* intravenous, *NHL* non-Hodgkin’s lymphoma, *NS* no significance, *os* oral solution, *SIADH* syndrome of inappropriate antidiuretic hormone secretion, *y/o* years old

Several case reports and retrospective studies describe the outcome of VIN when antifungal triazoles are co-administered [1, 69–76]. This interaction is mostly described in pediatric ALL patients but is also seen in adults (Table 3). Especially during chemotherapy induction, children are at increased risk of fungal infections due to neutropenia and corticosteroid administration; therefore, antifungal prophylaxis could be worth considering [69]. This interaction was first described in 1995 where 4 out of 14 adults experienced severe neurotoxicity during vincristine induction therapy and antifungal prophylaxis with itraconazole [72]. The prescribed itraconazole dose in this report was 400 mg/day; however, the same outcome has been observed and reported in several other cases treated with lower doses (Table 3). The study compared neurotoxic complications with a previous series of 460 ALL patients treated with an identical cytostatic regimen and found that the incidence of VIN enlarged and the symptoms were more severe. This severely aggravated neurotoxicity is also described by others [70, 75]; in addition, CNS toxicity has been recorded in 30% of the patients receiving itraconazole [76].

As mentioned, vincristine is metabolized by CYP3A. Fluconazole and itraconazole inhibit the action of CYP3A, and the former is far less potent than itraconazole [109]. Furthermore, itraconazole inhibits the P-glycoprotein efflux pump, a vincristine transporter, resulting in higher intracellular vincristine concentrations [110]. Thus, coadministration of vincristine and itraconazole decreases the metabolism of vincristine to a greater extent than coadministration with fluconazole, causing notably enhanced neurotoxicity. Consistently, three studies did not observe significant differences in neuropathy upon fluconazole treatment [69, 71, 73], whereas six independent studies report higher incidence and severe neuropathy upon itraconazole treatment [1, 70, 72, 74–76] (Table 3).

A study by Yang et al. found that coadministration of vincristine with itraconazole or voriconazole resulted in a significantly higher incidence of vincristine-associated adverse effects than in patients treated with fluconazole and control patients only treated with vincristine [1]. Additionally, they found that the incidence of VIN was significantly higher in itraconazole-treated patients compared with patients treated with voriconazole.

Numerous drugs are capable of CYP3A inhibition/induction and consequently have the potential to interfere with vincristine. Protease inhibitor-based antiretroviral therapy, such as ritonavir, causes excessive neurotoxicity when co-administered with vinblastine, another vinka alkaloid, due to CYP3A4 inhibition [77, 78]. Furthermore, a study from a 1996 report on drug–drug interactions between colony-stimulating factors and vincristine indicated that the underlying mechanisms for the observed induced neuropathy is not thoroughly studied; to our knowledge, the mechanism not been reported to date [79].

Drug interactions should always be kept in mind when administering more than one drug but especially when administering toxic drugs, such as chemotherapy. When using vincristine, which is almost completely metabolized by the liver, it is important to be aware of concomitant use of a drug that is also metabolized by CYP3A. Fluconazole should be preferred over both voriconazole and itraconazole for antifungal prophylaxis or treatment given that itraconazole especially has the potential to cause severe neurotoxicity.

### Prevention and treatment

Vincristine-induced neuropathy can be momentary and reversible; however, it is occasionally irreversible [87, 89]. Regardless, neuropathy may consequently necessitate dose-reduction or cessation, thus reducing the treatment efficacy and potentially shortening the overall survival time. Another perspective is that neuropathy can significantly impact the QOL [94], and neuropathy severity does not necessarily correlate with how much it affects the patient [30]. These problems can potentially be overcome by identifying substances with the ability to prevent and/or treat neuropathy.

Several agents have been suggested and tested for prevention and treatment of VIN; however, the results have not been promising. Currently, convincing evidence of useful pharmacologic interventions is lacking.

A study provides information on the beneficial effects from bracing and physical therapy to prevent fixed contractures/surgery when suffering from peroneal nerve palsy [80]. An older study from 1992 proposed Org 2766, a corticotropin (4–9) analogue, as a means to ameliorating vincristine neuropathy and additionally observed higher neurotoxicity in the placebo group [81]. Another study did not find Org 2766 to be neuroprotective [85].

Gabapentin has been tested both as prophylaxis and treatment in pediatric ALL; however, no conclusions could be made other than gabapentin did not prevent the recurrence of neuropathic pain [44]. Moreover, analgesic adjuvants given to reduce neuropathy symptoms did not show adequate prophylactic efficacy [49].

Pyridoxine (vitamin B6) and pyridostigmine (acetylcholinesterase inhibitor) have been used in the treatment of different types of VIN. Treatment of 4 ALL patients with pyridoxine and pyridostigmine for sensorimotor polyneuropathy resulted in full recovery 1–2 weeks after treatment initiation [82]. In agreement, a case study presented an ALL patient who on day 61 was diagnosed with bilateral cranial nerve VII and XII palsy and subsequently treated with intravenous pyridoxine, and complete recovery of symptoms was achieved after 2 weeks of treatment [19]. In both reports, uncertainty remained regarding whether pyridoxine/pyridostigmine was responsible for the resolution as it could have been a consequence of vincristine discontinuation.

To evaluate potential preventive effects of glutamic acid, a randomized study was conducted on 94 children diagnosed with hematological malignancies and 12 with Wilms tumor who received either glutamic acid (1.5 g/day divided into 3 doses) or placebo [10]. They found a statistically significant difference between the two groups when assessing paresthesia, patellar/Achilles reflexes, and frequency of constipation but not until the third and fourth visit, suggesting that glutamic acid may improve tolerance of vincristine. In contrast, a larger randomized study in which 200 children with ALL or NHL and 50 children with Wilms tumor or rhabdomyosarcoma received either preventive glutamic acid (250 mg capsules 3 times a day, body surface area < 1 m^2^ = 1 capsule, > 1 m^2^ = 2 capsules) or placebo did not find a statistically significant difference in any parameters [83]. Another similar amino acid, glutamine, has shown possible beneficial effects on sensory neuropathy and self-reported QOL [84]. The study included 31 children with hematological malignancies and 18 children with nonhematological malignancies, which were only analyzed as one group.

Due to the high prevalence of VIN, it would be of great significance to find possible prevention or treatment measures. Unfortunately, it has not been possible to find a substance with this property in current studies. Many of the substances are only tested a few times and in small studies, but glutamine could display potential if further studied.

## Discussion

This systematic review identified a total of 71 studies addressing the circumstances concerning VIN in hematological patients. Although this subject obviously contains many aspects, almost none of the findings are clear-cut. In general, more research is needed given that VIN can necessitate treatment alterations and possibly last for a long period of time, significantly impacting QOL.

In addition to a cumulative dose, this systematic review did not find consistency for any clinical predictors potentially due to differences in cohort sizes, vincristine doses and cycles, and neuropathy assessment tools. In addition, several studies include patients with different cancer diagnoses, which potentially could introduce a bias given that vincristine interacts differently with cancers [46, 88]. Consequently, further studies in larger settings are urgently warranted to fully elaborate the impact of these clinical parameters.

Caution is important with patients already suffering from CMT; however, the rareness and common time of diagnosis of this condition make it troublesome. A simple self-reporting checklist, which is not expensive or time-consuming for hospital staff, could be helpful to identify undiagnosed CMT patients before administrating vincristine [50].

With the greater attention towards molecular importance, the existence of molecular predictors present promise. Studies of molecular predictors differed in genotype detection technique and neuropathy detection method and grading, which complicated the general cross-comparison of the studies. Despite these differences, variants in the vincristine metabolizer *CYP3A5* leading to reduced CYP3A5 expression were consistently reported as a risk factor for VIN (Table 2). Noteworthy, the frequency of *CYP3A5* variants varies between ethnicities; consequently, the reported incidence of VIN is higher in Caucasians than African–Americans. Thus, ethnicity could be used as a risk factor for VIN; however, it is important to emphasize that it is only a surrogate for CYP3A5 and that individual genotype assessment is preferable. In addition to vincristine metabolizers, genetic alterations in genes involved in vincristine pharmacodynamics were associated with VIN. A SNP in CEP72 showed great promise as predictor of VIN in two independent ALL cohorts [62, 68] but failed stratification in a Spanish ALL cohort [63]. Molecular risk factors clearly contribute to a better understanding of vincristine neurotoxicity and are potentially useful in the identification of individuals at higher risk, which could optimize and personalize vincristine dosing, leading to maximum response while minimizing the risk of long-term neuropathy.

The results of drug–drug interference as a cause of increased risk of VIN were very consistent and independent of cohort size, diagnosis and methodology. Coadministration with itraconazole and to lesser extent voriconazole is an important aspect of VIN cause and worsening (Table 3). Based on the findings included in this review, fluconazole appears safer when concomitantly used with vincristine and should be preferred as antifungal prophylaxis and treatment. Given that the mechanism of this interaction involves inhibition of CYP3A in the liver, it could be advisable to be aware of other strong CYP3A inhibitors or inducers.

Unfortunately, neither substances for preventing VIN nor treatment options have been discovered to date, resulting in substitution, reduction or discontinuation as the only method of handling neurotoxicity. Glutamic acid potentially prevents or at least improves tolerance of vincristine in one study.

In general, various neuropathy detection methods and grading systems are used for all aspects of neuropathy examined in this review, making it very difficult to compare studies and interpret the results between studies. Although the WHO had great intentions to create a standardized tool of assessing information about neuropathy, it obviously did not succeed or persist as a useful rating scale since only two of the 71 included studies used this tool. Nerve conduction studies are thought to be the most accurate method of quantifying VIN but are time consuming, costly and discomforting to be a standard VIN measurement in clinical practice. In contrast, subjective measures introduce the risk of interobservational variability. With the many new neurotoxicity scores used today, it is yet again of importance to agree on but not necessarily standardize which score fits which situation best and thereby makes it possible to compare studies.

## Conclusion

In conclusion, clinical parameters did not show convincing potential as predictors of VIN. In contrast, molecular markers, including polymorphisms in the hepatic vincristine metabolizer CYP3A5, displayed great promise in predicting increased incidence and severity of neuropathy; however, further studies are warranted to assess its use in treatment prediction. Consistently, antifungal drugs, such as itraconazole and voriconazole, inhibit the action of CYP3A5; consequently, coadministration of vincristine and these drugs decreases vincristine metabolism, leading to enhanced neuropathy side effects. More true markers of both clinical and molecular origin may emerge if consistency in VIN detection and reporting increases through the use of standardized neuropathy assessment tools and grading scales.

## Electronic supplementary material

Below is the link to the electronic supplementary material.

Supplementary material 1 (PPTX 859 kb)

Supplementary material 2 (DOCX 14 kb)
