# Supplementary material for: Aspects of vincristine-induced neuropathy in hematologic malignancies: a systematic review
Source: Cancer Chemother Pharmacol. 2019 Jun 18;84(3):471–85. doi: 10.1007/s00280-019-03884-5 (PMC6682573; doi:10.1007/s00280-019-03884-5)
Supplement: Supplementary file 1 — Supplementary material 1 (PPTX 859 kb) [file 280_2019_3884_MOESM1_ESM.pptx]

## Slide 1
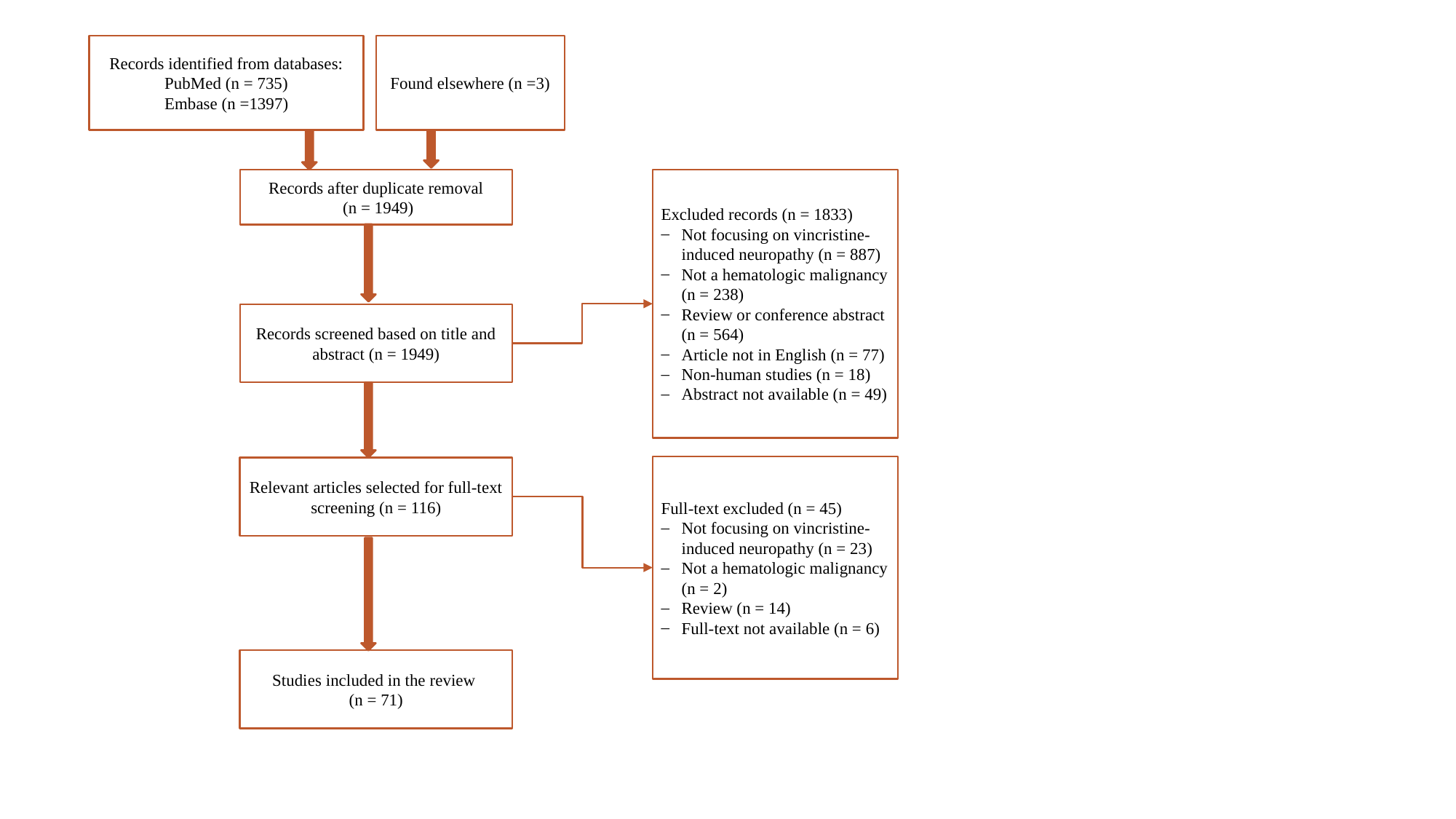

Records identified from databases:
PubMed (n = 735)
Embase (n =1397)
Found elsewhere (n =3)
Records after duplicate removal
 (n = 1949)
Excluded records (n = 1833)
Not focusing on vincristine-induced neuropathy (n = 887)
Not a hematologic malignancy (n = 238)
Review or conference abstract (n = 564)
Article not in English (n = 77)
Non-human studies (n = 18)
Abstract not available (n = 49)
Records screened based on title and abstract (n = 1949)
Full-text excluded (n = 45)
Not focusing on vincristine-induced neuropathy (n = 23)
Not a hematologic malignancy (n = 2)
Review (n = 14)
Full-text not available (n = 6)
Relevant articles selected for full-text screening (n = 116)
Studies included in the review
(n = 71)
