# Supplementary material for: Aspects of vincristine-induced neuropathy in hematologic malignancies: a systematic review
Source: Cancer Chemother Pharmacol. 2019 Jun 18;84(3):471–85. doi: 10.1007/s00280-019-03884-5 (PMC6682573; doi:10.1007/s00280-019-03884-5)
Supplement: Supplementary file 2 — Supplementary material 2 (DOCX 14 kb) [file 280_2019_3884_MOESM2_ESM.docx]

**Supplementary Table 1**. Search strategy in PubMed and Embase

**Database PubMed** **Embase**

**Search terms** #1: “Vincristine” [Mesh] #1: ‘vincristine’/exp

#2: Vincristine* #2: vincristin*:ti,ab,kw

#3: Oncovin* #3: ‘vincristine sulfate’/exp

#4: ”Vinblastine” [Mesh] #4: oncovin*:ti,ab,kw

#5: Vinblastin* #5: ‘vinblastine’/exp

#6: #1 OR #2 OR #3 OR #4 OR #5 #6: vinblastin*:ti,ab,kw

#7: neuropath* #7: #1 OR #2 OR #3 OR #4 OR #5

#8: ”Neurotoxicity Syndromes” [Mesh:noexp] OR #6

#9: neurotoxicit* #8: ‘peripheral neuropathy’/exp

#10: sensory impairment* #9: (peripheral NEAR/2

#11: #7 OR #8 OR #9 OR #10 (neuropath* OR toxicit*)):ti,ab,kw

#12: “Hematologic Neoplasms” [Mesh] #10: #8 OR #9

#13: “Lymphoma” [Mesh] #11: leukemia:ti,ab,kw OR

#14: “Leukemia” [Mesh] lymphoma*:ti,ab,kw

#15: hematolog* OR haematolog* OR myeloma:ti,ab,kw

#16: leukemia OR lymphoma* #12: ‘hematologic malignancy’/exp

#17: “Hematologic Diseases” [Mesh] #13: #11 OR #12

#18: “Multiple Myeloma” [Mesh] #14: #7 AND #10 AND #13

#19: myeloma*

#20: #12 OR #13 OR #14 OR #15 OR #16 Filters:

OR #17 OR #18 OR #19 NOT ‘conference abstract’/lt

#21: #6 AND #11 AND #20 NOT ‘nonhuman’/de

No filters
